# Combining topic models with bipartite blockmodelling to uncover the multifaceted nature of social capital

**DOI:** 10.1371/journal.pone.0253478

**Published:** 2021-06-18

**Authors:** Jef Vlegels, Stijn Daenekindt

**Affiliations:** Department of Sociology, Ghent University, Ghent, Belgium; University of Sao Paulo, BRAZIL

## Abstract

In this paper, we seek to identify the existing conceptualisations and applications of social capital contained in the literature, as well as how these are used and combined across and within research fields. Our analytical approach presents a unique combination of topic models and bipartite blockmodelling, enabling us to analyse both the content and structures of a large collection of academic texts. In particular, this allows us to: (a) summarise the content in relation to a variety of topics; and (b) uncover the structure, with diverse text subsets engaging differently with these topics. Our analysis of all of the 11,975 articles on Web of Science that address ‘social capital’ demonstrates that these can be reduced to nine distinct topic clusters and six article clusters. Specifically, we identify the multifaceted nature of the social-capital metaphor and show that there are clear variations in how it is deployed in different bodies of literature. Finally, by mapping the diverse conceptualisations and applications of social capital in a network, we propose a tool for identifying future research opportunities for those interested in novel social-capital treatments in their field.

## Introduction

The concept of social capital has increasingly gained currency in scientific research over recent decades, and continues to be of significant interest to scholars from a wide range of disciplines. This has generated an impressive volume of work that invokes the concept to answer an extensive range of questions. Extant research demonstrates that social capital–in short, the connections among social actors–plays an important role in, e.g., social cohesion, access to information, political participation, civic engagement, transaction costs, opportunistic behaviour, risk and uncertainty insurance, and collective action [1–7]. These widespread uses and applications reflect the recognition by many researchers that social capital is a fundamental feature of social lives; or, in Coleman’s [8] words, it highlights the ‘appropriability’ of social connections for different purposes.

At the same time, this appropriability is associated with an often-expressed critique that social capital is a handy, catch-all, umbrella concept [9]. Sceptics argue that social capital is ‘a wonderfully elastic term’ [10] that means ‘many things to many people’ [11]. Some authors also argue that the ‘social’ in social capital makes no contribution to the discussion, since in their interpretations ‘capital’ is inherently social [12]. Several scholars are pessimistic about the lack of an unequivocal definition, and regard the concept as a ‘for-all and cure-all sociological term’ [13], which has taken on ‘a circus-tent quality’ [14]. This critique addresses the conceptually, theoretically and operationally fragmented nature of social capital, which can even be seen in the first publications on the concept [13, 15]. In line with Lin & Erickson [13], however, we are less pessimistic and argue that the diversity of applications and understandings of the concept are indicative of its omnipotence.

The diversity in the conceptualisations of social capital was already visible when it was introduced into the social sciences. In the late 1980s, when the concept gained traction, it was based on the classic work of Pierre Bourdieu [16], James Coleman [8] and Robert Putnam [5], each of whom argued that social relations are associated with personal and societal advantage. The precise conceptualisations of these scholars differ substantially, however. Bourdieu defines social capital as ‘the sum of actual or potential resources that are linked to the possession of a durable network of more or less institutionalised relationships of mutual acquaintance and recognition–in other words, to membership in a group’ [16]. Social networks are thus at the heart of his definition, with more specific opportunities and advantages related to being a member of a certain group. To Bourdieu, the richness of social capital depends on the size of the network and the volume of capital available in the connections, with the outcome mainly a personal instrumental value, e.g., economic and social benefits.

James Coleman, on the other hand, defines social capital by its function [1]: he considers it to be a valuable resource available to an actor through his or her social relationships [17]. It arises, according to Coleman, from two different entities: a social structure, and the actions of individuals who are part of it. The relationships between actors in this structure create obligations, expectations, trust and information flows. This web of relations also makes it feasible to achieve goals that would be impossible without the network. Coleman, therefore, effectively bridges both the individual and the collective, seeing social capital as ‘a capital asset for the individual’ that is also constructed from ‘social structural resources’ [18].

In contrast to the sociologists Bourdieu and Coleman, Robert Putnam is a political scientist and has arguably played the most prominent role in the popularisation of the social capital concept. According to Putnam, ‘social capital refers to features of social organization, such as trust, normal and networks, that can improve the efficiency of society by facilitating coordinated actions’ [5]. He discusses the concept mainly in the context of civic engagement, as both the number of civic organisations and the participation in them are an indication of a specific society’s social capital. Civic engagement fosters robust norms, reciprocity and trust, according to Putnam, which has a positive effect on the public good [19]. Consequently, social capital in this conceptualisation is a collective property and cannot be transformed into a private good.

Dasgupta & Serageldin [20] argue that the work of these three authors and, more importantly, the fundamental differences between their conceptualisations, became the seedbed for the current multifaceted nature of the concept. Indeed, an abundance of definitions, theoretical advances and operationalisations emerged after the publication of the initial work of Bourdieu, Coleman and Putnam, with a number of scholars trying to produce a common definition and analytical approach that combines the insights discussed in one general model [e.g., 21–23]. Unfortunately, however, these efforts have commonly led to vague, confusing definitions and no consensus on a general conceptualisation [13].

Linked to the differences between the definitions of Bourdieu, Coleman and Putnam, we argue that the multifaceted nature of the concept of social capital also ensues from its use in an abundance of applications across various research fields [17]. Indeed, authors in different fields have often developed a specific definition tailored to their particular research focus [2, 3, 24, 25]. As a result, the range of available conceptualisations expands with almost every additional publication. We, therefore, argue that investigations of the multifaceted nature of social capital must go hand in hand with a study of the fields in which the concept is applied.

The availability of various conceptualizations of the concept *as such* is not a problem because, as indicated earlier, it can be considered an indicator of its potential and its ‘appropriability’. Therefore, instead of trying to produce a common definition, we believe that it is far more valuable to understand the different conceptualisations of the concept of social capital and to contextualize these by situating them in the wider literature. We believe that shedding light on this is valuable in four ways. First, it allows researchers to better understand how the concept has evolved from the classic definitions of Bourdieu, Putnam and Coleman. Indeed, retracing the intellectual lineage of the concept social capital sheds light on which aspects of the classic definitions withstood time and which elements have been introduced. Second, by mapping the different conceptualizations in different fields of study, researchers can identify opportunities for future research. Moreover, such a mapping can be a first step in the cross-fertilization of ideas from different application fields and, in this way, inspire future theoretical developments. Third, and related to the second point, our bird’s-eye view of the different conceptualizations of social capital can avoid future parallel (and redundant) developments in different fields of application. Finally, by getting a grip on the various conceptualisations of social capital and how they differ from each other, we hope to avoid that the social capital concept evolves into in a meaningless, and therefore useless, container concept. By improving our understanding on how conceptualizations differ from each other, and in which situations a specific conceptualization of social capital is valuable, we contribute to the hands-on usability of the theoretical concept for different practical research problems.

In this article, we aim to shed light on the diversity of both the conceptualisations of social capital and the fields in which it is applied. Previous work has tried to address this fragmentation by providing overviews of existing research [7, 24, 26–31]. However, we identify two weaknesses in this approach. First, extant studies tend to focus on one specific discipline, disentangling the different conceptualisations within a particular disciplinary field or subfield and summarising the main findings relevant to it [21]. As a consequence, these studies do not do justice to either the diverse ways social capital is conceptualised across disciplines or the various applications in which it is being deployed. The result is redundant parallel developments and the pursuit of research questions that have already seen progress in other fields. Second, and related to this, extant work runs the risk of being affected by the author’s perspective, which may hamper the quality and conclusions of the research. More specifically, the selective reporting of outcomes and studies is a known potential issue of systematic reviews [32].

In this paper, we apply a bottom-up, data-driven approach to overcome these problems and address the following two research questions: (a) What are the existing conceptualisations and fields of application of social capital across scientific disciplines? (b) How are these different conceptualisations and fields employed and combined within and across diverse areas of research? Our analysis does not involve a traditional systematic review methodology. Instead, we analysed all the articles on Web of Science that address social capital and investigated their content by combining two statistical methods capable of managing such big data: topic modelling and bipartite blockmodelling. In this way, our contribution to the literature is twofold: (a) our results provide scholars interested in social capital with an exhaustive overview of different understandings and applications. This helps with the identification of gaps in the literature and, therefore, opportunities for future research; and (b) we demonstrate the potential of combining topic models with bipartite blockmodelling, a technique stemming from network analysis. Topic models have been employed on scientific publications before [33–36]; combining network analysis with topic models is also fairly common [37–39]. However, the network properties analysed in these studies are usually metadata related to the documents, e.g., whether publications cite one another [38, 39]. In contrast, we use network analysis to examine the relationships between the topics in the corpus. Indeed, we introduce a new approach by applying a bipartite blockmodelling procedure to the results of topic models (i.e., the per-document-per-topic matrix). By retaining the bipartite structure of this matrix, instead of projecting it into one mode, we are able to detect clusters of topics and clusters of articles simultaneously. This allows us to demonstrate how combinations of the different social capital conceptualisations and fields of application are typically employed. In this way, our paper also makes a methodological contribution by highlighting both the compatibility of the two methods, as well as how they can be combined to gain insights into the structure and contents of large collections of texts.

## Materials and methods

### Data

Our focus in this study was on articles on Web of Science (WoS). Although other research databases are, of course, available (e.g., Scopus and Google Scholar), WoS is the dominant research citation database [40]. Moreover, consideration of articles from more inclusive databases would also capture peripheral publications, adding further noise to our findings.

**Fig 1:**
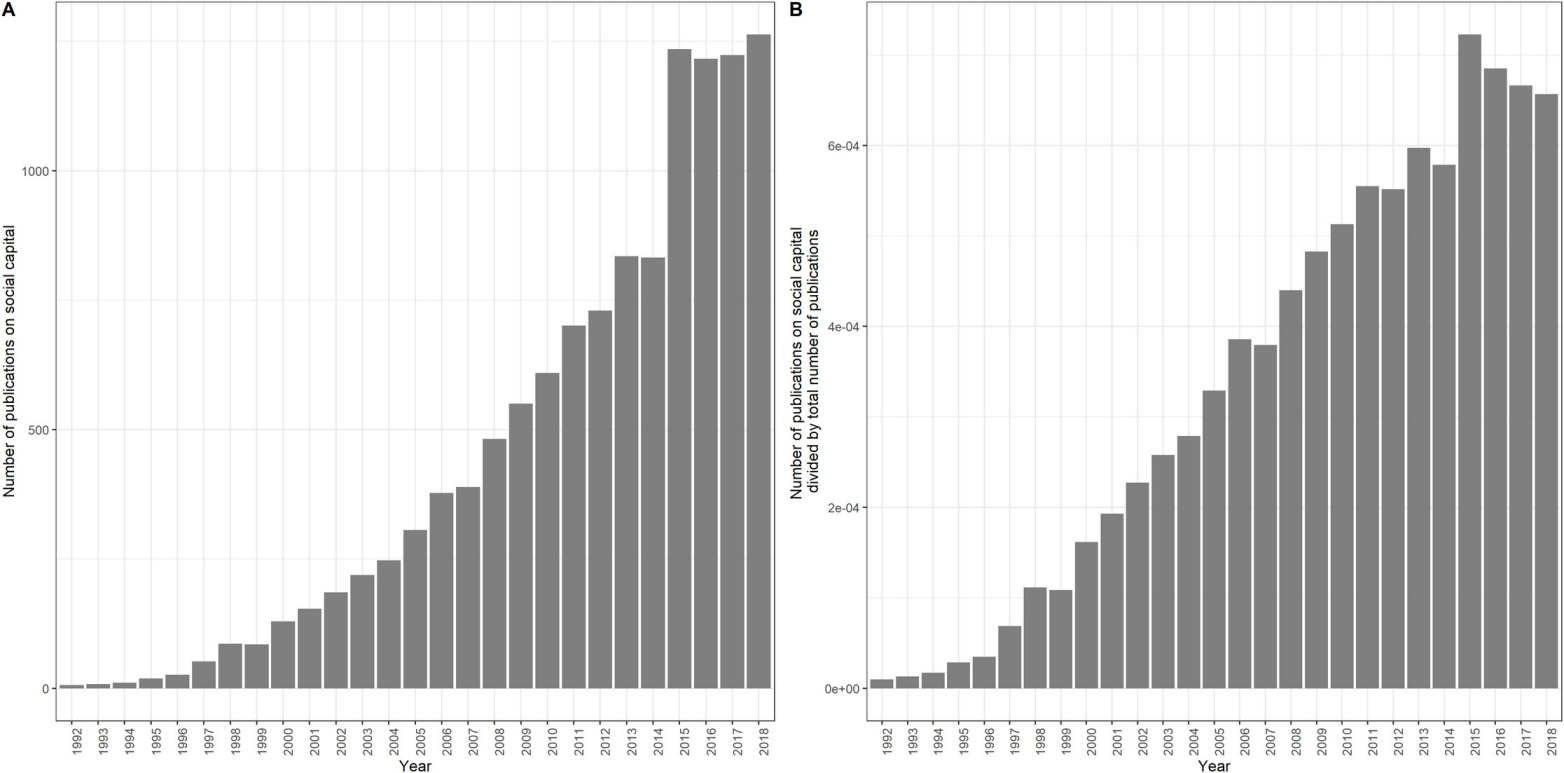
Absolute and relative frequency of publications on social capital by the year of publication.

We used a search query (i.e., the ‘TS’ query on WoS) to identify all the articles that included the term ‘social capital’ in their Title, Abstract, Keywords or Keywords Plus fields. More specifically, our query on 1 January, 2019 was as follows: <TS = “social capital”; document type = “Article”>. This produced an initial raw selection of 12,168 articles, the abstracts of which we then analysed. The advantage of focusing on abstracts is that they represent a summary of an article by its author. In addition, abstracts are (compared to complete or specific sections of articles) publicly available and more comparable across journals and disciplines in terms of the stylistic requirements [34, 35]. Given the high number of documents produced by our search, analysing our corpus using human coding was unfeasible. We therefore conducted a type of automated content analysis using topic models (see below for more details). As topic models perform poorly with short documents, we removed 184 articles with fewer than 50 words [41]. We also detected nine duplicates that were subsequently excluded from the dataset. Ultimately, the corpus used for the analyses contained 11,975 articles. Fig 1 shows the distribution of these papers across the different years of publication. Fig 1A is based on the absolute frequencies. Fig 1B plots relative frequencies by dividing the number of documents by the total number of publications in WoS for each year. The histograms confirms an increasing trend in terms of the number of publications on social capital from 1992 to 2018, both in absolute as in relative numbers.

We pre-processed the abstracts to prepare them for the analysis. All the letters were lowercased, and punctuation marks, numbers and stop-words (e.g., ‘and’; ‘which’) were removed. We also accounted for differences between UK and US spelling. We identified multiword expressions using quanteda [42] and manually selected the relevant bigrams (two-word pairs) and trigrams (three-word groups). In this way, we consider, e.g., the following multiword expressions: ‘civil_society’, ‘health_care’, ‘labor_market’, and ‘european_social_survey’. Next, we used Porter’s algorithm [43] to apply the linguistic stemming technique. Stemming reduces complexity by removing the ends of words in order to decrease the total number of unique words. As an example, the words ‘political’, ‘politics’ and ‘politician’ share the stem ‘polit’, and so we replaced them with ‘polit’. Infrequently used terms were removed from the dataset, as these do not contribute to achieving an understanding of patterns in a corpus. Words that appeared in fewer than 1% of the documents were removed [cf. 44].

### Topic models

The first step of our analysis involved the use of topic models, which enable the discovery of latent topics in large collections of text. The method uses patterns in co-occurrences of words in documents to reveal latent themes (i.e., topics) across documents, and models each document as a mix of multiple topics [45]. Our focus was on two sets of probabilities: *per-topic-per-word* and *per-document-per-topic*. The former tells us which words are most likely to appear for each topic, while the latter shows the proportions of the topics for each document. By examining the highest probabilities for a topic, we were then able to seek out the documents that exemplified each of them.

In considering our research questions, we first estimated models with five, 10 and 15 topics. Then, each author independently investigated the most likely words for each topic and the abstracts that loaded highest on the topics. We concluded that the ideal ‘level of granularity of the view into the data’ [46] ranged from 10 to 15. Consequently, we fitted models that estimated 11, 12, 13 and 14 topics. Again, both authors independently inspected each model. Finally, based on this model-selection procedure, we chose a model that estimated 13 topics.

### Bipartite blockmodelling

In the second part of the analysis, we applied a bipartite blockmodelling procedure to the per-document-per-topic matrix. Bipartite blockmodelling stems from network analysis and allows the simultaneous identification of clusters of articles and clusters of topics; article clusters contain articles that have similar relationships to the identified topic clusters. Conversely, topic clusters contain topics that have similar relationships to the identified article clusters. This approach helped us to determine the ways in which different article clusters engaged with the different topics. It also enabled us to identify the characteristic properties of each topic cluster, which relates to their position in the network of topic clusters and article clusters. As an example, a topic cluster might be relatively exclusively linked to a specific article cluster (i.e., specialisation), versus a position whereby it is widely associated with different article clusters (i.e., diversification). This methodology helped us to identify how different conceptualisations and application fields are used and combined within and across different research disciplines (cf., our second research question).

As a starting point for this analysis, we interpreted the per-document-per-topic probability matrix as a bipartite network of articles in the first mode and topics in the second mode. Also known as two-mode network, a bipartite network can be defined as a graph: *G* = {*U, V, E*}, where *U* and *V* are two disjointed sets of nodes (i.e., articles and topics) and *E* = {(*u_i_, v_j_*): *u_i_*∈*U, v_j_*∈*V*} describes the links between them (i.e., the probabilities between the articles and topics according to the topic model). As *U* and *V* are disjointed, there are no connections between nodes from the same mode.

Bipartite networks can be projected into a one-mode network by linking nodes from the first (second) mode that share a relationship with a node from the second (first) [47]. However, this inevitably results in a significant loss of information, as the un-projected mode’s nodes—and their associated attributes—disappear [48]. Consequently, we retained the bipartite network for our subsequent analyses and deployed specific bipartite techniques.

The bipartite per-document-per-topic probability matrix was used to set up a bipartite blockmodelling procedure [49]. This process differs from other procedures like hierarchical clustering or k-means, as it clusters both modes of the per-document-per-topic matrix, i.e., the document and the topic mode, *simultaneously*. The basic idea behind this method is to permutate the rows and columns of the matrix in such a way that they are re-organised into homogeneous blocks (i.e., empty or complete). A disadvantage of this simultaneous process is that it is very expensive computationally. Nevertheless, the recent block expectation maximisation algorithm (BEM) and new software packages (i.e., the blockcluster package in R) make it a workable procedure, even for relatively large datasets.

Our per-document-per-topic matrix was prepared by dichotomising the probabilities prior to running the blockmodelling. This is a necessary step because the topic-model procedure means that the sum of the proportions in one row (i.e., the sum of the topic loadings for each article) always equals one in the per-document-per-topic matrix. Consequently, the probabilities in the matrix are not randomly distributed and cannot be used for a Gaussian bi-clustering procedure. According to the criterion adopted by Curran et al. [37], we dichotomised the probabilities in each column by assigning a value of one if the probability was twice as high as the expected loading when the topics were equally important for an article (i.e., a loading of (1/13)*2). The resulting matrix, which served as the basis for the bi-clustering procedure, had 11,975 rows (articles) and 13 columns (topics). This is as a sparse matrix, as 84.38% of the elements had a value of zero.

We ran the blockmodelling procedure for all possible combinations of clusters in the article and the topic modes, and used the integrated complete likelihood (ICL) criteria to select the optimal number per row (article) and column (topic). In our case, the highest ICL value (ICL = -62,073.95) was related to the solution of six article clusters and nine topic clusters.

## Results

### Topics

The topic model results, more specifically the main interpretation of the 13 detected topics, are discussed in this section. The top words for each topic are presented in Table 1. These words were identified using the FREX (FRequency and EXclusivity) measure, which produces the mean of a word’s rank in terms of its frequency and exclusivity and has been shown to be more interpretable than the often-used frequency-based summaries [50]. The topics were ranked based on their proportion in the corpus: for example, the relative prevalence of topic 1, which was most prominent in the corpus, was 14.1%. Topic 13, on the other hand, was the least prominent, with a presence of only 3.8%.

**Table 1:** Presentation of the topics identified by our model.

Topic	Proportion	Top words^a^ (the first three, in italics, are used as topic labels)
1	14.10%	*Political*, *concept*, *public*, civic, argue, society, volunteering
2	10.50%	*Trust*, *level*, *rates*, *general*, income, membership, estimate
3	9.90%	*Networks*, *bridging*, *tie*, online, bonding, media, information
4	9.70%	*Sustainable*, *farmers*, *rural*, food, local, adaptation, environmental
5	8.10%	*Program*, *care*, *community*, disaster, services, HIV, healthcare
6	8.00%	*Growth*, *entrepreneurs*, *capital*, migrants, economic, economy, entrepreneurship
7	8.00%	*Firms*, *innovation*, *capabilities*, companies, strategic, performance, industry
8	7.10%	*Sharing*, *knowledge*, *organizational*, employees, team, learning, leadership
9	5.70%	*Immigrants*, *ethnic*, *university*, career, college, minority, class
10	5.50%	*Older*, *age*, *depression*, adjusted, social support, risk, symptoms
11	5.20%	*Neighbourhood*, *health*, *wellbeing*, physical, mental, self-rated, poor
12	4.40%	*City*, *urban*, *people*, young, housing, youth, crime
13	3.80%	*School*, *family*, *children*, parents, teachers, education, adolescents

^a^ We replaced the stems with the most common unstemmed word to improve readability.

We interpreted each topic based on the top words presented in Table 1 and close readings of the 10 highest loading articles for each topic (see the S1 File for a list of these articles). It is important to note that a topic may refer to a specific conceptualisation of social capital and/or a specific field of application. In relation to our research question, we were interested in both of these and interpret the two elements of each topic in detail below.

In topic 1, ‘political, concept, trust’, the concept of social capital is used to refer to norms that bind people together in communities and create trust between them. In line with this, studies often cite De Tocqueville [51] and Putnam [47]. The way social capital is interpreted here resembles the way it is understood in topic 11, ‘neighbourhood, health, wellbeing’, although topic 1 clearly engages more with the theoretical history of the concept and applies it to the study of civil society and democracy.

Topic 2, ‘trust, level, rates’, focuses on what has been referred to as the cognitive side of social capital, i.e., norms of generalised trust [52]. These studies generally rely on survey research, as also suggested by the top words ‘estimate’ and ‘rates’, and operationalise generalised trust as the belief that ‘most people can be trusted’. This research applies this understanding of social capital to a variety of outcomes; that is, this topic does not engage clearly with one specific field of application.

Topic 3, ‘networks, bridging, tie’, focuses on studies conceptualising social capital as access to resources and so examines the extent to which people can benefit from network participation. The focus is on applications in online networks (e.g., Facebook, Twitter). More specifically, the central question here concerns how participation in online networks can influence social capital.

Topic 4, ‘sustainable, farmers, rural’, relates to research that applies social capital in analyses of climate change, and includes studies examining how communities can implement strategies to manage this. While this research often contains the term social capital in the title or abstract, explanations of the role of the concept are very limited and it is rarely referred to in the body of these articles. In line with this, these studies do not define social capital and their reflections on the concept do not go much further than, e.g., concluding that improving it could have benefits for a community.

The focus in topic 5, ‘program, care, community’, is on interventions to help people manage problems with their (mental) health. These interventions are aimed at, e.g., reducing stigma and increasing empowerment. Studies in this application field stress the role of social capital, which, in their conceptualisation, refers to mutual support and care. In that sense, this research discusses interventions to increase social capital, e.g., by organising self-help groups.

Topic 6, ‘growth, entrepreneurs, capital’, relates to the role of social capital in understanding economic development. It is conceptualised here as social networks and trust, which is considered to be vital for coordination and collaboration. These studies regard social capital as a facilitator of economic transactions, meaning that its application field can be situated in economic-development research. In addition, and as indicated by the top word ‘migrants’, this work often examines the role of migrants/migration when it comes to understanding how it can help with the accumulation of (social) capital.

Topic 7, ‘firms, innovation, capabilities’, applies the concept of social capital to innovation in firms, as indicated by the two top words, ‘firms’ and ‘innovation’. The conceptualisation of social capital in these studies is also specific to this endeavour; that is, social capital refers here to the resources available via a firm’s relationships with external actors.

Topic 8, ‘sharing, knowledge, organizational’, concerns social capital as it relates to knowledge-sharing. This is embedded in a strand of research that examines knowledge-sharing in the context of collaboration within teams, which these studies suggest is a vital prerequisite for good performance. In this way, this work attempts to achieve a better understanding of which type of social climate in a team leads to the best sharing of knowledge. Social capital is conceptualised here as the social interactions between the different members of a team, and as the best way to produce the optimal social climate.

Topic 9, ‘immigrants, ethnic, university’, conceptualises social capital as the resources rooted in an individual’s social network. The focus is on applications relating to career advancement. The interest of these studies is also on how the advantages of social capital in an individual’s career differ between socio-demographic groups, in particular affecting those with a migratory background.

Topic 10, ‘older, age, depression’, applies social capital to the issue of depression; more specifically, to how a lack of social capital makes people more susceptible to depression. Social capital is conceptualised in these studies as ‘network size’ and so is therefore interpreted very numerically (i.e., the *number* of people in one’s network). As indicated by the top words ‘older’ and ‘age’, particular attention is paid to the elderly.

Topic 11, ‘neighbourhood, health, wellbeing’, centres on neighbourhood social capital and is embedded in research that examines neighbourhood characteristics to explain differences between them and between individuals. Of the possible neighbourhood characteristics, there is particular interest in neighbourhood social capital, which means that social capital is conceptualised here as the degree of social cohesion. This interpretation is particularly employed to explain differences in health outcomes and quality of life, which is also indicated by the topic words used in these studies (e.g., ‘health’ and ‘wellbeing’).

Topic 12, ‘city, urban, people’, focuses on applications in urban settings, with scholars employing the topic to examine, e.g., youth care, crime and drug use. These studies conceptualise social capital as the quality and degree of social attachment and, more generally, how it relates to experiences of social support.

Topic 13, ‘school, family, children’, focuses on the intergenerational transmission of educational advantages. These studies use family social capital to understand differences in school outcomes between students from diverse social backgrounds. Social capital is conceptualised as the relationship between parents and children, and the extent of the former’s involvement in the latter’s schooling.

Table 2 presents a summary of our interpretations of each topic, with particular attention paid to two aspects: the way social capital is conceptualised within each topic *and* the field of application.

**Table 2:** Summary of the interpretations of the topic models.

Topic	Topic label	Interpretation	
		Conceptualisation	Field of application
1	*Political*, *concept*, *public*	Reciprocity and trustworthiness	Democracy, civil society
2	*Trust*, *level*, *rates*, *general*	Generalised trust	/
3	*Networks*, *bridging*, *tie*	Online social capital	Online network participation
4	*Sustainable*, *farmers*, *rural*	/	Adaption to climate change
5	*Program*, *care*, *community*	Mutual support and care	Coping with mental problems
6	*Growth*, *entrepreneurs*, *capital*	Social network and trust	Economic development
7	*Firms*, *innovation*, *capabilities*	Firms’ external resources	Innovation in firms
8	*Sharing*, *knowledge*, *organizational*	Social interaction between team members	Knowledge sharing
9	*Immigrants*, *ethnic*, *university*	Resources rooted in social networks	Career advantages
10	*Older*, *age*, *depression*	Network size	Depression
11	*Neighbourhood*, *health*, *wellbeing*	Neighbourhood social capital	Health, well-being, quality of life
12	*City*, *urban*, *people*	Experiencing social support	Urban setting
13	*School*, *family*, *children*	Family social capital	Academic achievement

### Bipartite blockmodelling

**Fig 2:**
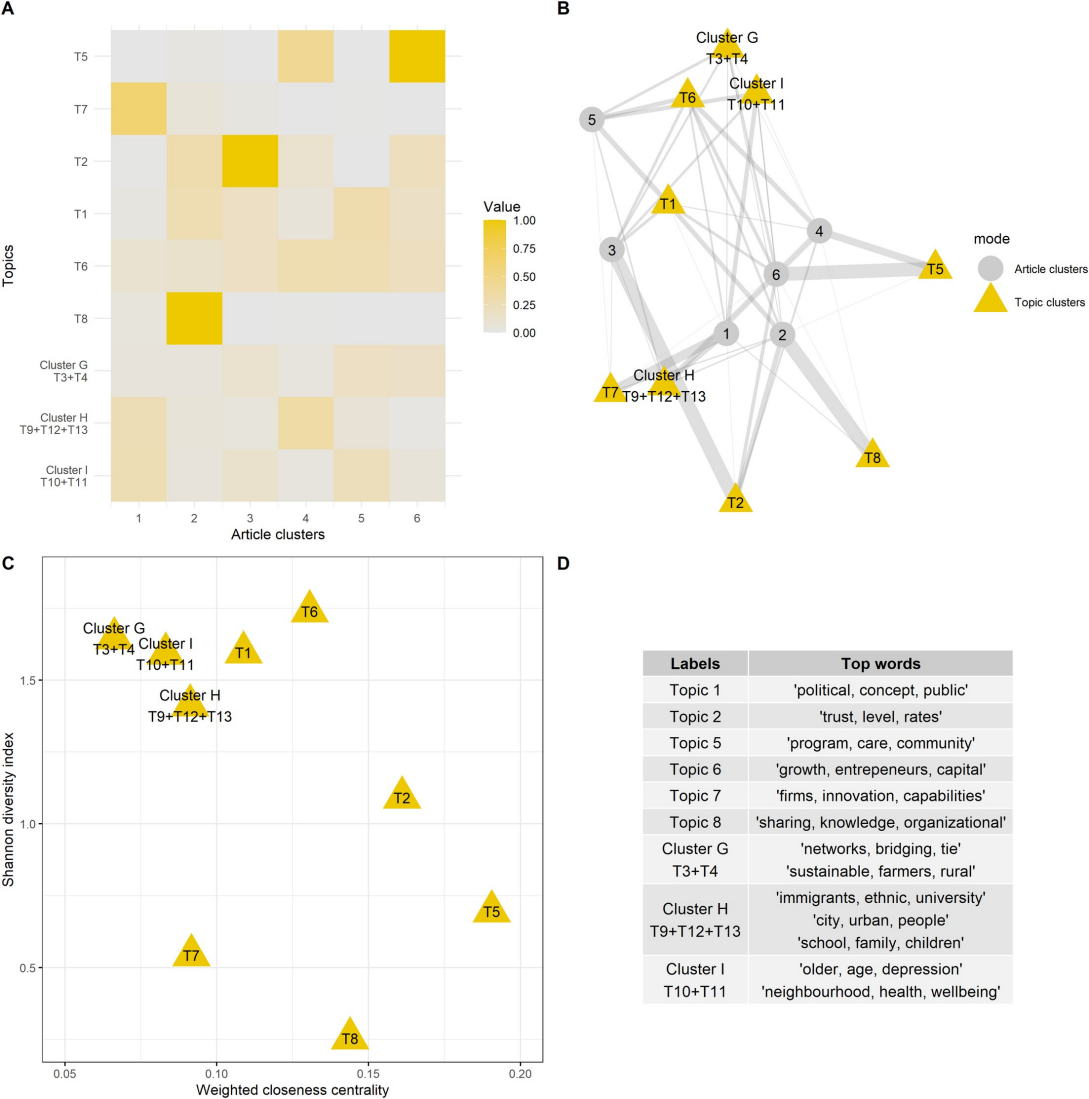
Results of the bipartite clustering solution.

The second part of this results section focuses on the bipartite blockmodelling process and the relationship between the article and topic clusters. Fig 2, and more specific the heatmap in Fig 2A, depicts how the clustering procedure described in section 3.4 reduces the 13 topics to nine topic clusters and the 11,975 articles to six article clusters.

Topics were clustered together if they had a similar relationship to the detected article clusters. This may have occurred for two reasons. First, a topic cluster may contain topics that are related in substantial ways. Consider, e.g., cluster H, which combines the topics ‘immigrants, ethnic, university’, ‘city, urban, family’ and ‘school, family, children’. These all have related interpretations of social capital, with the focus on how interpersonal connections lead to personal advantages. Although these understandings are slightly different, this appears to be of no consequence in studies involving these topics, because they generally consider these three approaches simultaneously. Likewise, topic cluster I combines ‘older, age, depression’ and ‘neighbourhood, health, wellbeing’, both of which deal with personal (physical or mental) health issues. However, there is some difference between them, with ‘neighbourhood, health, wellbeing’ focused more on neighbourhood social capital, and ‘older, age, depression’ on personal-network size.

Second, clustering two or more topics together does not necessarily imply that they have similar content; it only shows that they are typically ‘used’ (or not used) in a similar way in the different article clusters, which leads to the clustered topic solution. The best example of this is cluster G, which combines the topics ‘networks, bridging, tie’ and ‘sustainable, farmers, rural’. These have fairly different interpretations and fields of application, with the former discussing online social capital and the latter situated in the literature on climate change. However, what ties them together is their relative indifference with respect to the different article clusters, with which there are no strong associations and only relatively weak connections to article clusters three, five and six.

The six article clusters can be interpreted in an equivalent manner, with consideration only now given to the colour-shade patterns in Fig 2A’s heatmap columns, which represent the relationships between the article clusters and the topic clusters. The model reduces the original corpus of 11,975 documents to six article clusters that have similar relationships to the topic clusters. This means that, although we detected quite diverse interpretations of the social capital concept (i.e., 13 different topics), publications on the concept are limited in terms of whether and how these are considered. In theory, many more combinations of these 13 different topics are possible, but our analysis demonstrates that the majority are very uncommon in current research.

Fig 2B visualises the heatmap as a network to aid interpretability. This makes the position of these clusters in the network and the relationships between the topic and article clusters easier to understand. The plot depicted is a Fruchterman-Reingold force-directed graph of the heatmap in Fig 2A, where the size of the edges is proportional to the proportions in the cells of the heatmap, and the shape and the colour of the nodes are dependent on the network mode [53]. A force-directed graph pulls nodes that have similar connections closer to each other, and pushes away those that are more dissimilar. This makes it easier to detect relationships between nodes that would otherwise be difficult to uncover.

The network in Fig 2A shows how the topic ‘growth, entrepreneurs, capital’ and topic-cluster G and topic-cluster I are situated close together. Indeed, their relationships with the article clusters are similar. Specifically, their ties are relatively equally dispersed towards the article clusters. This pattern is notably different than, e.g., the tie structure of topic-cluster H, which is less dispersed and dominated more by specific relationships to a few of the article clusters. This is even more the case for topic 5 ‘program, care, community’ and topic 2 ‘trust, level, rates’; meanwhile, the tie structure of topic 1 ‘political, concept, public’ seems more similar to the pattern for topic 6 ‘growth, entrepreneurs, capital’ and topic-cluster G and topic-cluster I.

Similar patterns emerge when focusing on the article clusters. Fig 2A and 2B show that article-clusters 2, 3 and 6 are predominantly linked to one specific topic. More specifically, article-cluster 2 is focused on ‘sharing, knowledge, organizational’, article-cluster 3 on ‘trust, level, rates’, and article-cluster 6 on ‘program, care, community’. All the other links between these clusters are much less pronounced, demonstrating the specialized nature of article-clusters 2, 3 and 6. In contrast, because article-clusters 1, 4 and 5 have a much more dispersed pattern of ties, it is clear that their focus is on several different topics and topic clusters at the same time. Their different positions in the network in Fig 2B reflect the fact that their tie pattern is distinct. More specifically, article-cluster 1 concerns topics and topic clusters that conceptualise and apply social capital at the level of the individual. This involves personal networks in the topic ‘firms, innovation, capabilities’, as well as in ‘school, family, children’, ‘immigrants, ethnic, university’, ‘city, urban, people’, and the clusters on ‘neighbourhood, health, wellbeing’ and ‘older, age, depression’. Article-cluster 4, on the other hand, is more focused on higher level conceptualisations of social capital, which treat it as a characteristic of the community. This involves topics such as ‘program, care, community’, ‘trust, level, rates’, ‘growth, entrepreneurs, capital’, and the same topic-clusters, i.e., ‘school, family, children’, ‘immigrants, ethnic, university’ and ‘city, urban, people’. Finally, in article-cluster 5, the topic cluster ‘neighbourhood, health, wellbeing’ and ‘older, age, depression’ is combined with the topics ‘political, concept, public’ and ‘growth, entrepreneurs, capital’ and the topic-clusters ‘networks, bridging, tie’ and ‘sustainable, farmers, rural’.

Fig 2C supports this positional interpretation, providing a centrality and a diversity index for each topic cluster. Weighted-closeness centrality is an indicator for the average distance to all the other nodes in the network [54, 55]. It can be seen that a central topic, e.g., ‘program, care, community’, is typically used by article clusters that also encompass several other topics, thereby reducing the average distance to all those that are not part of the cluster. The Shannon diversity captures the diversity in the ties of a topic cluster. A low diversity index, e.g., for ‘sharing, knowledge, organizational’, indicates that this topic has a very dominant relationship with one, or a limited number of, article clusters. This is thus an indication that this topic is considered in only a particular set of articles (article-cluster 2). A very diverse topic (e.g., ‘growth, entrepreneurs, capital’), on the other hand, is examined directly by many of the article clusters. It is important to note that these two measures are not necessarily related positively; a topic cluster can have, e.g., high diversity and a relatively low average distance. This is the case for topic-cluster G.

## Discussion and conclusion

In some ways, the social capital metaphor is a victim of its own success. The multifaceted nature of the concept is the result of a vast and enthusiastic research-community interest that emerged right from the time of its introduction in the late 1980s. Previous attempts to reign in the fragmented nature of the concept have tended to focus on one specific discipline and, in this way, run the risk of bias arising from a particular author’s perspective. Consequently, in this study, we employed a bottom-up, data-driven approach to produce an exhaustive overview of the different conceptualisations of social capital used by researchers in various application fields.

Our findings provide those who are interested in social capital with an exhaustive map that shows the existing interpretations, but also how different bodies of literature engage with them. This is important as it shows how conceptualizations of social capital have evolved from the classic definitions of Bourdieu, Coleman and Putnam to a very diverse set of conceptualizations. The common ground is still clear: connections among social actors matter. Nonetheless, we find great variety between conceptualizations in which social actors and which connections are considered relevant, and why these actors and connections are relevant. These findings confirm the multifaceted nature of the social capital concept and its appropriability in different settings. At the same time, the common ground in the different conceptualizations show that the concept has not evolved into a meaningless helicopter concept.

A second important take-away from our mapping is that while some conceptualizations seem omnipresent, others are rather exclusively linked to a specific research field. This has two important consequences. First, it highlights future opportunities for researchers interested in new applications of the social capital metaphor. Specifically conceptualizations with a low diversity and limited centrality offer opportunities for researchers to apply them in new application fields. Second, this overview of existing conceptualizations in the different research fields might help interested researchers in avoiding parallel developments in different unconnected fields of applications.

In addition to providing insights into the various interpretations and uses of the social capital metaphor, our work also makes a methodological contribution. Our analysis employs a two-step approach consisting of a combination of topic models and bipartite blockmodelling. This combination shows great potential when it comes to understanding both the content and structure in large collections of texts. This is achieved by detecting the different topics discussed in a document and, subsequently, identifying clusters of documents that differ in terms of the content highlighted by the topic model.

In this article, we presented a bird-eye’s view of all research that relies on the concept of social capital. Our analysis offers readers insights in the various existing conceptualizations of the concept and how these conceptualizations differ from each other. In addition, we present a contextualization of each conceptualization by situating them in the wider literature. E.g., which disciplinary fields are dominated by which conceptualization? In this way, we hope that this article contributes to future research on social capital by helping researchers understand each other. Indeed, instead of developing one ‘master conceptualisation’ of social capital, we believe it is more important that researchers understand other conceptualisations and, in this way, are able to learn from research that departs from other conceptualisations. Researchers using the social capital metaphor may speak different languages—as there may be substantial differences in their conceptualisation of social capital—and we hope that this article prevents them from getting lost in translation.

## Supporting information

S1 File The ten highest-loading articles for each topic. (DOCX)
