# Supplementary material for: Combining topic models with bipartite blockmodelling to uncover the multifaceted nature of social capital
Source: PLoS One. 2021 Jun 18;16(6):e0253478. doi: 10.1371/journal.pone.0253478 (PMC8213168; doi:10.1371/journal.pone.0253478)
Supplement: S1 File — (DOCX) [file pone.0253478.s001.docx]

**S1 File. The ten highest-loading articles for each topic.**

| Topic 1 |  |
| --- | --- |
| 1 | Kim, S. (2010). On Korean dual civil society: Thinking through Tocqueville and Confucius. *Contemporary Political Theory*, 9(4), 434-457. |
| 2 | Hadiz, V. R. (2004). Decentralization and democracy in Indonesia: A critique of neo‐institutionalist perspectives. *Development and Change*, 35(4), 697-718. |
| 3 | Walters, W. (2002). Social capital and political sociology: re-imagining politics?. *Sociology*, 36(2), 377-397. |
| 4 | Ivana, G. I. (2017). Fake it till you make it: imagined social capital. *The Sociological Review*, 65(1), 52-66. |
| 5 | Foley, M. W., & Edwards, B. (1998). Beyond Tocqueville: civil society and social capital in comparative perspective: editors' introduction. *American Behavioral Scientist*, 42(1): 5-20. |
| 6 | Kuah, K. E. (2016). Singapore state and the emergence of Buddhist welfarism. *Ageing International*, 41(4), 335-349. |
| 7 | Spanjer, M. (2017). The Happiness Machine, or How to be an Archaeologist in a Changing World. *The Historic Environment: Policy & Practice*, 8(3), 206-211. |
| 8 | Tollebeek, J. (2015). The Use of History in Belgium and the Netherlands, 1945–65: Presentism and Historicism in the Work of Jan Romein, Pieter Geyl, and Leopold Flam. *Dutch Crossing*, 39(1), 54-73. |
| 9 | Ester, P., & Vinken, H. (2003). Debating civil society: On the fear for civic decline and hope for the internet alternative. *International Sociology*, 18(4), 659-680. |
| 10 | Swart, I. (2017). Social capital, religious social capital and the missing element of religious ritual. *Religion and Theology*, 24(3-4), 221-249. |
| Topic 2 |  |
| 1 | Lindberg, E., & Farkas, G. M. (2016). Much ado about nothing? A multilevel analysis of the relationship between voluntary associations' characteristics and their members' generalized trust. *Journal of Civil Society*, 12(1), 33-56. |
| 2 | Rönnerstrand, B. (2016). Contextual generalized trust and immunization against the 2009 A (H1N1) pandemic in the American states: A multilevel approach. *SSM-Population Health*, 2, 632-639. |
| 3 | Nagaoka, K., Fujiwara, T., & Ito, J. (2012). Do income inequality and social capital associate with measles-containing vaccine coverage rate?. *Vaccine*, 30(52), 7481-7488. |
| 4 | Richey, S. (2010). The impact of corruption on social trust. *American Politics Research*, 38(4), 676-690. |
| 5 | Huisman, M., & Oldehinkel, A. J. (2009). Income inequality, social capital and self-inflicted injury and violence-related mortality. *Journal of Epidemiology & Community Health*, 63(1), 31-37. |
| 6 | Pericoli, F. M., Pierucci, E., & Ventura, L. (2015). The impact of social capital on consumption insurance and income volatility in the UK: evidence from the British Household Panel Survey. *Review of Economics of the Household*, 13(2), 269-295. |
| 7 | Kawachi, I., Kennedy, B. P., Lochner, K., & Prothrow-Stith, D. (1997). Social capital, income inequality, and mortality. *American Journal of Public Health*, 87(9), 1491-1498. |
| 8 | Han, S. (2015). Social capital and subjective happiness: Which contexts matter?. *Journal of Happiness Studies*, 16(1), 241-255. |
| 9 | Sulemana, I. (2015). An empirical investigation of the relationship between social capital and subjective well-being in Ghana. *Journal of Happiness Studies*, 16(5), 1299-1321. |
| 10 | Beilmann, M., & Realo, A. (2012). Individualism-collectivism and social capital at the individual level. *Trames: A Journal of the Humanities and Social Sciences*, 16(3), 205. |
| Topic 3 |  |
| 1 | Jin, C. H. (2015). The role of Facebook users’ self-systems in generating social relationships and social capital effects. *New Media & Society*, 17(4), 501-519. |
| 2 | Riedl, C., Köbler, F., Goswami, S., & Krcmar, H. (2013). Tweeting to feel connected: A model for social connectedness in online social networks. *International Journal of Human-Computer Interaction*, 29(10), 670-687. |
| 3 | Quinn, K. (2016). Contextual social capital: Linking the contexts of social media use to its outcomes. *Information, Communication & Society*, 19(5), 582-600. |
| 4 | Phua, J., Jin, S. V., & Kim, J. J. (2017). Uses and gratifications of social networking sites for bridging and bonding social capital: A comparison of Facebook, Twitter, Instagram, and Snapchat. *Computers in Human Behavior*, 72, 115-122. |
| 5 | Domahidi, E., Festl, R., & Quandt, T. (2014). To dwell among gamers: Investigating the relationship between social online game use and gaming-related friendships. *Computers in Human Behavior*, 35, 107-115. |
| 6 | Meng, J., Williams, D., & Shen, C. (2015). Channels matter: Multimodal connectedness, types of co-players and social capital for Multiplayer Online Battle Arena gamers. *Computers in Human Behavior*, 52, 190-199. |
| 7 | Matzat, U., & Sadowski, B. M. (2015). Access to specific social resources across different social media: divergent consequences of the time spent with new contacts. *Information, Communication & Society*, 18(10), 1139-1157. |
| 8 | Su, C. C., & Chan, N. K. (2017). Predicting social capital on Facebook: The implications of use intensity, perceived content desirability, and Facebook-enabled communication practices. *Computers in Human Behavior*, 72, 259-268. |
| 9 | Weiqin, E. L., Campbell, M., Kimpton, M., Wozencroft, K., & Orel, A. (2016). Social capital on Facebook: The impact of personality and online communication behaviors. *Journal of Educational Computing Research*, 54(6), 747-786. |
| 10 | Liu, Y., Venkatanathan, J., Goncalves, J., Karapanos, E., & Kostakos, V. (2014). Modeling what friendship patterns on Facebook reveal about personality and social capital. *ACM Transactions on Computer-Human Interaction*, 21(3), 1-20. |
| Topic 4 |  |
| 1 | Uy, N., Takeuchi, Y., & Shaw, R. (2011). Local adaptation for livelihood resilience in Albay, Philippines. *Environmental Hazards*, 10(2), 139-153. |
| 2 | Li, S., Juhász-Horváth, L., Pintér, L., Rounsevell, M. D., & Harrison, P. A. (2018). Modelling regional cropping patterns under scenarios of climate and socio-economic change in Hungary. *Science of the Total Environment*, 622, 1611-1620. |
| 3 | Alam, G. M., Alam, K., & Mushtaq, S. (2016). Influence of institutional access and social capital on adaptation decision: Empirical evidence from hazard-prone rural households in Bangladesh. *Ecological Economics*, 130, 243-251. |
| 4 | Dolan, A. H., & Walker, I. J. (2006). Understanding vulnerability of coastal communities to climate change related risks. *Journal of Coastal Research*, 1316-1323. |
| 5 | McDowell, J. Z., & Hess, J. J. (2012). Accessing adaptation: Multiple stressors on livelihoods in the Bolivian highlands under a changing climate. *Global Environmental Change*, 22(2), 342-352. |
| 6 | Larson, L. R., Lauber, T. B., Kay, D. L., & Cutts, B. B. (2017). Local government capacity to respond to environmental change: Insights from towns in New York State. *Environmental Management*, 60(1), 118-135. |
| 7 | Johnston, M., & Hesseln, H. (2012). Climate change adaptive capacity of the Canadian forest sector. *Forest Policy and Economics*, 24, 29-34. |
| 8 | Ruiz-Agudelo, C. A., Bonilla-Uribe, O. D., & Páez, C. A. (2015). The vulnerability of agricultural and livestock systems to climate variability: using dynamic system models in the Rancheria upper basin (Sierra Nevada de Santa Marta). *Journal on Protected Mountain Areas Research and Management*, 7, 50-60. |
| 9 | Jara-Rojas, R., Bravo-Ureta, B. E., & Díaz, J. (2012). Adoption of water conservation practices: A socioeconomic analysis of small-scale farmers in Central Chile. *Agricultural Systems*, 110, 54-62. |
| 10 | Akiefnawati, R., Villamor, G. B., Zulfikar, F., Budisetiawan, I., Mulyoutami, E., Ayat, A., & van Noordwijk, M. (2010). Stewardship agreement to reduce emissions from deforestation and degradation (REDD): Case study from Lubuk Beringin's Hutan Desa, Jambi Province, Sumatra, Indonesia. *International Forestry Review*, 12(4), 349-360. |
| Topic 5 |  |
| 1 | Chung, B., Corbett, C. E., Boulet, B., Cummings, J. R., Paxton, K., McDaniel. S., Mercier, S. O., Franklin, C., Mercier, E., Jones, L., Collins, B.E., Koegel, P., Duan, N., Wells, K.B., Glik, D. (2006). Talking Wellness: a description of a community-academic partnered project to engage an African-American community around depression through the use of poetry, film, and photography. *Ethnicity and Disease*, 16(1), S1. |
| 2 | Judd, J., & Keleher, H. (2014). Building health promotion capacity in a primary health care workforce in the Northern Territory: some lessons from practice. *Health Promotion Journal of Australia*, 24(3), 163-169. |
| 3 | Stang, I., & Mittelmark, M. B. (2010). Intervention to enhance empowerment in breast cancer self‐help groups. *Nursing Inquiry*, 17(1), 47-57. |
| 4 | Lubega, M., Musenze, I. A., Joshua, G., Dhafa, G., Badaza, R., Bakwesegha, C. J., & Reynolds, S. J. (2013). Sex inequality, high transport costs, and exposed clinic location: reasons for loss to follow-up of clients under prevention of mother-to-child HIV transmission in eastern Uganda–a qualitative study. *Patient Preference and Adherence*, 7, 447. |
| 5 | Perez‐Brumer, A. G., Reisner, S. L., McLean, S. A., Silva‐Santisteban, A., Huerta, L., Mayer, K. H., Sanchez, J., Clark, J. L., Mimiaga, M. J., Lama, J. R. (2017). Leveraging social capital: multilevel stigma, associated HIV vulnerabilities, and social resilience strategies among transgender women in Lima, Peru. *Journal of the International AIDS Society*, 20(1), 21462. |
| 6 | Vargas, R. B., Ryan, G. W., Jackson, C. A., Rodriguez, R., & Freeman, H. P. (2008). Characteristics of the original patient navigation programs to reduce disparities in the diagnosis and treatment of breast cancer. *Cancer*, 113(2), 426-433. |
| 7 | Kakietek, J., Geberselassie, T., Manteuffel, B., Ogungbemi, K., Krivelyova, A., Bausch, S.,  Rodriguez-García, R., Bonnel, R., N'Jie, N., Fruh, J., Gar, S. (2013). It takes a village: community-based organizations and the availability and utilization of HIV/AIDS-related services in Nigeria. *AIDS Care*, 25(sup1), S78-S87. |
| 8 | Fletcher, S., Mullett, J., & Beerman, S. (2014). Value of a regional family practice residency training program site: Perceptions of residents, nurses, and physicians. *Canadian Family Physician*, 60(9), e447-e454. |
| 9 | Skovdal, M., Magutshwa-Zitha, S., Campbell, C., Nyamukapa, C., & Gregson, S. (2013). Community groups as ‘critical enablers’ of the HIV response in Zimbabwe. *BMC Health Services Research*, 13(1), 195. |
| 10 | Harris, F. M., Maxwell, M., O’Connor, R. C., et al. (2013). Developing social capital in implementing a complex intervention: a process evaluation of the early implementation of a suicide prevention intervention in four European countries. *BMC Public Health*, 13(1), 158. |
| Topic 6 |  |
| 1 | Li, Y., Wang, X., Westlund, H., & Liu, Y. (2015). Physical Capital, Human Capital, and Social Capital: The Changing Roles in C hina's Economic Growth. *Growth and change*, 46(1), 133-149. |
| 2 | Wahba, J., & Zenou, Y. (2012). Out of sight, out of mind: Migration, entrepreneurship and social capital. *Regional Science and Urban Economics*, 42(5), 890-903. |
| 3 | Åkesson, L. (2016). Multi‐sited accumulation of capital: Cape Verdean returnees and small‐scale business. *Global Networks*, 16(1), 112-129. |
| 4 | England, R. W. (2000). Natural capital and the theory of economic growth. *Ecological Economics*, 34(3), 425-431. |
| 5 | Batjargal, B., & Liu, M. (2004). Entrepreneurs’ access to private equity in China: The role of social capital. *Organization Science*, 15(2), 159-172. |
| 6 | Baumgartner, D., Pütz, M., & Seidl, I. (2013). What kind of entrepreneurship drives regional development in European non-core regions? A literature review on empirical entrepreneurship research. *European Planning Studies*, 21(8), 1095-1127. |
| 7 | Batabyal, A. A., & Nijkamp, P. (2010). Richard Florida’s creative capital in a trading regional economy: A theoretical investigation. *The Annals of Regional Science*, 44(2), 241-250. |
| 8 | Fritsch, M., & Storey, D. J. (2014). Entrepreneurship in a regional context: Historical roots, recent developments and future challenges. *Regional Studies*, 48(6), 939-954. |
| 9 | Shamaileh, A. (2018). Barriers to Financial Institutional Development: A Preliminary Theoretical Exploration of Social Capital, Growth and Institutional Development. *Economics Bulletin*, 38(1), 186-195. |
| 10 | De Haas, H. (2010). The internal dynamics of migration processes: A theoretical inquiry. *Journal of Ethnic and Migration Studies*, 36(10), 1587-1617. |
| Topic 7 |  |
| 1 | Li, Y., Zhang, Y., & Zheng, S. (2016). Social capital, portfolio management capability and exploratory innovation: evidence from China. *Journal of Business & Industrial Marketing*, 31(6), 794-807. |
| 2 | Oke, A., Prajogo, D. I., & Jayaram, J. (2013). Strengthening the innovation chain: The role of internal innovation climate and strategic relationships with supply chain partners. *Journal of Supply Chain Management*, 49(4), 43-58. |
| 3 | Ruiz-Ortega, M. J., Parra-Requena, G., & Garcia-Villaverde, P. M. (2016). Do territorial agglomerations still provide competitive advantages? A study of social capital, innovation, and knowledge. *International Regional Science Review*, 39(3), 259-290. |
| 4 | Kim, K. T., Lee, J. S., & Lee, S. Y. (2017). The effects of supply chain fairness and the buyer’s power sources on the innovation performance of the supplier: A mediating role of social capital accumulation. *Journal of Business & Industrial Marketing*, 32(7), 987-997. |
| 5 | Basuil, D. A., & Datta, D. K. (2017). Value creation in cross-border acquisitions: The role of outside directors' human and social capital. *Journal of Business Research*, 80, 35-44. |
| 6 | Hsieh, M. H., & Tsai, K. H. (2007). Technological capability, social capital and the launch strategy for innovative products. *Industrial Marketing Management*, 36(4), 493-502. |
| 7 | Dost, M., Arshad, M., & Afsar, B. (2018). The influence of entrepreneurial orientation on types of process innovation capabilities and moderating role of social capital. *Entrepreneurship Research Journal*, 8(4). |
| 8 | Wu, L. Y., & Wang, C. J. (2007). Transforming resources to improve performance of technology-based firms: A Taiwanese Empirical Study. *Journal of Engineering and Technology Management*, 24(3), 251-261. |
| 9 | Wu, W., Liu, Y., & Chin, T. (2018). The effect of technology management capability on new product development in China’s service-oriented manufacturing firms: a social capital perspective. *Asia Pacific Business Review*, 24(2), 212-232. |
| 10 | Kohtamäki, M., Partanen, J., & Möller, K. (2013). Making a profit with R&D services—The critical role of relational capital. *Industrial Marketing Management*, 42(1), 71-81. |
| Topic 8 |  |
| 1 | Zhang, L., & Cheng, J. (2015). Effect of knowledge leadership on knowledge sharing in engineering project design teams: the role of social capital. *Project Management Journal*, 46(5), 111-124. |
| 2 | Huang, C. C., & Jiang, P. C. (2012). Exploring the psychological safety of R&D teams: An empirical analysis in Taiwan. *Journal of Management & Organization*, 18(2), 175-192. |
| 3 | Yuan, M., Zhang, X., Chen, Z., Vogel, D. R., & Chu, X. (2009). Antecedents of coordination effectiveness of software developer dyads from interacting teams: an empirical investigation. *IEEE Transactions on Engineering Management*, 56(3), 494-507. |
| 4 | Hau, Y. S., Kim, B., Lee, H., & Kim, Y. G. (2013). The effects of individual motivations and social capital on employees’ tacit and explicit knowledge sharing intentions. *International Journal of Information Management*, 33(2), 356-366. |
| 5 | Lin, C. P. (2011). Modeling job effectiveness and its antecedents from a social capital perspective: A survey of virtual teams within business organizations. *Computers in Human Behavior*, 27(2), 915-923. |
| 6 | Chen, M., & Qi, X. (2015). Members’ satisfaction and continuance intention: a socio-technical perspective. *Industrial Management & Data Systems*, 115(6), 1132-1150. |
| 7 | Prieto-Pastor, I., Martín-Pérez, V., & Martín-Cruz, N. (2018). Social capital, knowledge integration and learning in project-based organizations: a CEO-based study. Journal of Knowledge Management, 22(8), 1803-1825. |
| 8 | Brown, S., Chen, L., & O’Donnell, E. (2017). Organizational opinion leader charisma, rolemodeling, and relationships. *International Journal of Organizational Analysis*, 25(1), 80-102. |
| 9 | Avgar, A. C. (2010). Negotiated capital: conflict, its resolution, and workplace social capital. *International Journal of Conflict Management*, 21(3), 236-259. |
| 10 | Lee, H., Park, J., & Lee, J. (2013). Role of leadership competencies and team social capital in IT services. *Journal of Computer Information Systems*, 53(4), 1-11. |
| Topic 9 |  |
| 1 | Parks-Yancy, R., DiTomaso, N., & Post, C. (2006). The social capital resources of gender and class groups. *Sociological Spectrum*, 26(1), 85-113. |
| 2 | Abrahamsen, B., & Drange, I. (2015). Ethnic minority students’ career expectations in prospective professions: Navigating between ambitions and discrimination. *Sociology*, 49(2), 252-269. |
| 3 | McDonald, S., Lin, N., & Ao, D. (2009). Networks of opportunity: Gender, race, and job leads. *Social Problems*, 56(3), 385-402. |
| 4 | Dumangane Jr, C. (2017). The significance of faith for Black men's educational aspirations. *British Educational Research Journal*, 43(5), 875-903. |
| 5 | Abada, T., & Tenkorang, E. Y. (2009). Gender differences in educational attainment among the children of Canadian immigrants. *International Sociology*, 24(4), 580-608. |
| 6 | Parks-Yancy, R. (2006). The effects of social group membership and social capital resources on careers. *Journal of Black Studies*, 36(4), 515-545. |
| 7 | Museus, S. D., & Mueller, M. K. (2018). Understanding how key institutional agents provide Southeast Asian American students with access to social capital in college. *Journal of College Student Development*, 59(2), 192-209. |
| 8 | Parks-Yancy, R. (2012). Interactions into opportunities: Career management for low-income, first-generation African American college students. *Journal of College Student Development*, 53(4), 510-523. |
| 9 | Chua, V., Mathews, M., & Loh, Y. C. (2016). Social capital in Singapore: Gender differences, ethnic hierarchies, and their intersection. *Social Networks*, 47, 138-150. |
| 10 | Wells, R. S., Seifert, T. A., Padgett, R. D., Park, S., & Umbach, P. D. (2011). Why do more women than men want to earn a four-year degree? Exploring the effects of gender, social origin, and social capital on educational expectations. *The Journal of Higher Education*, 82(1), 1-32. |
| Topic 10 |  |
| 1 | Langille, D., Asbridge, M., Kisely, S., & Wilson, K. (2012). Risk of depression and multiple sexual risk-taking behaviours in adolescents in Nova Scotia, Canada. *Sexual Health*, 9(3), 254-260. |
| 2 | Fu, C., Yang, F., & Mao, Z. (2018). Factors associated with cognitive decline among elderly in Wuhan, China living along living with children. *Southeast Asian Journal of Tropical Medicine and Public Health*, 49(4), 696-706. |
| 3 | Sakuraya, A., Imamura, K., Inoue, A., Tsutsumi, A., Shimazu, A., Takahashi, M., Totsuzaki, T., & Kawakami, N. (2017). Workplace social capital and the onset of major depressive episode among workers in Japan: a 3-year prospective cohort study. *J Epidemiol Community Health*, 71(6), 606-612. |
| 4 | Eguchi, H., Tsutsumi, A., Inoue, A., & Odagiri, Y. (2017). Psychometric assessment of a scale to measure bonding workplace social capital. *Plos one*, 12(6), e0179461. |
| 5 | Sakamoto, A., Ukawa, S., Okada, E., et al. (2017). The association between social participation and cognitive function in community‐dwelling older populations: Japan Gerontological Evaluation Study at Taisetsu community Hokkaido. *International Journal of Geriatric Psychiatry*, 32(10), 1131-1140. |
| 6 | Markkula, N., Härkänen, T., Nieminen, T., Pena, S., Mattila, A. K., Koskinen, S., Saarni, S. I., & Suvisaari, J. (2016). Prognosis of depressive disorders in the general population–results from the longitudinal Finnish Health 2011 Study. *Journal of Affective Disorders*, 190, 687-696. |
| 7 | Wang, C., Zhu, J., Cai, Y., Cui, D., Wang, Q., & Mao, Z. (2016). Community-based study of the relationship between social capital and cognitive function in Wuhan, China. *Asia Pacific Journal of Public Health*, 28(8), 717-724. |
| 8 | Amin, I. (2016). Social capital and sexual risk-taking behaviors among older adults in the United States. *Journal of Applied Gerontology*, 35(9), 982-999. |
| 9 | Markkula, N., Marola, N., Nieminen, T., Koskinen, S., Saarni, S. I., Härkänen, T., & Suvisaari, J. (2017). Predictors of new-onset depressive disorders–results from the longitudinal Finnish Health 2011 Study. *Journal of Affective Disorders*, 208, 255-264. |
| 10 | Rugulies, R., Hasle, P., Pejtersen, J. H., Aust, B., & Bjorner, J. B. (2016). Workplace social capital and risk of long-term sickness absence. Are associations modified by occupational grade?. *The European Journal of Public Health*, 26(2), 328-333. |
| Topic 11 |  |
| 1 | Mackenbach, J. D., Lakerveld, J., van Lenthe, F. J., Bárdos, H., Glonti, K., Compernolle, S., De Bourdeaudhuij, I., Oppert, J. M., Roda, C., Rutter, H., Brug, J., & Nijpels, G. (2016). Exploring why residents of socioeconomically deprived neighbourhoods have less favourable perceptions of their neighbourhood environment than residents of wealthy neighbourhoods. *Obesity Reviews*, 17, 42-52. |
| 2 | Drukker, M., & van Os, J. (2003). Mediators of neighbourhood socioeconomic deprivation and quality of life. *Social Psychiatry and Psychiatric Epidemiology*, 38(12), 698-706. |
| 3 | Maass, R., Kloeckner, C. A., Lindstrøm, B., & Lillefjell, M. (2016). The impact of neighborhood social capital on life satisfaction and self-rated health: A possible pathway for health promotion?. *Health & Place*, 42, 120-128. |
| 4 | Browne-Yung, K., Ziersch, A., & Baum, F. (2016). Neighbourhood, disorder, safety and reputation and the built environment: perceptions of low income individuals and relevance for health. *Urban Policy and Research*, 34(1), 17-38. |
| 5 | Zhang, Z., & Zhang, J. (2017). Perceived residential environment of neighborhood and subjective well-being among the elderly in China: A mediating role of sense of community. *Journal of Environmental Psychology*, 51, 82-94. |
| 6 | McAneney, H., Tully, M. A., Hunter, R. F., Kouvonen, A., Veal, P., Stevenson, M., & Kee, F. (2015). Individual factors and perceived community characteristics in relation to mental health and mental well-being. *BMC Public Health*, 15(1), 1237. |
| 7 | Wen, M., Browning, C. R., & Cagney, K. A. (2007). Neighbourhood deprivation, social capital and regular exercise during adulthood: A multilevel study in Chicago. *Urban Studies*, 44(13), 2651-2671. |
| 8 | Modie-Moroka, T. (2009). Does level of social capital predict perceived health in a community?—a study of adult residents of low-income areas of Francistown, Botswana. *Journal of Health, Population, and Nutrition*, 27(4), 462. |
| 9 | Poortinga, W. (2006). Perceptions of the environment, physical activity, and obesity. *Social Science & Medicine*, 63(11), 2835-2846. |
| 10 | Ohrnberger, J., Fichera, E., & Sutton, M. (2017). The relationship between physical and mental health: A mediation analysis. *Social Science & Medicine*, 195, 42-49. |
| Topic 12 |  |
| 1 | Sinkkonen, M. (2013). Attachment of young people to their home district. *Youth & Society*, 45(4), 523-544. |
| 2 | Van Audenhove, S., & Vander Laenen, F. (2017). Future expectations of young people leaving youth care in F landers: the role of personal and social capital in coping with expected challenges. *Child & Family Social Work*, 22(1), 256-265. |
| 3 | Macit, R. (2018). Becoming a drug dealer in Turkey. *Journal of Drug Issues*, 48(1), 106-117. |
| 4 | Baumont, C. (2009). Spatial effects of urban public policies on housing values. *Papers in Regional Science*, 88(2), 301-326. |
| 5 | Nasir, S., Rosenthal, D., & Moore, T. (2011). The social context of controlled drug use amongst young people in a slum area in Makassar, Indonesia. *International Journal of Drug Policy*, 22(6), 463-470. |
| 6 | Phillips, R. F. (2010). Initiatives to support disadvantaged young people: Enhancing social capital and acknowledging personal capital. *Journal of Youth Studies*, 13(4), 489-504. |
| 7 | Miller, J., McAuliffe, L., Riaz, N., & Deuchar, R. (2015). Exploring youths' perceptions of the hidden practice of youth work in increasing social capital with young people considered NEET in Scotland. *Journal of Youth Studies*, 18(4), 468-484. |
| 8 | Ilan, J. (2013). Street social capital in the liquid city. *Ethnography*, 14(1), 3-24. |
| 9 | Cheung, N. W., & Cheung, Y. W. (2006). Is Hong Kong experiencing normalization of adolescent drug use? Some reflections on the normalization thesis. *Substance Use & Misuse*, 41(14), 1967-1990. |
| 10 | Dangschat, J. S. (1994). Concentration of poverty in the landscapes of 'boomtown' Hamburg: The creation of a new urban underclass?. *Urban Studies*, 31(7), 1133-1147. |
| Topic 13 |  |
| 1 | Yang, H. (2017). The role of social capital at home and in school in academic achievement: The case of South Korea. *Asia Pacific Education Review*, 18(3), 373-384. |
| 2 | McNeal, R. B. (2015). Parent involvement and student performance: The influence of school context. *Educational Research for Policy and Practice*, 14(2), 153-167. |
| 3 | Durand, T. M. (2011). Latino parental involvement in kindergarten: Findings from the early childhood longitudinal study. *Hispanic Journal of Behavioral Sciences*, 33(4), 469-489. |
| 4 | Azmitia, M., Cooper, C. R., & Brown, J. R. (2009). Support and guidance from families, friends, and teachers in Latino early adolescents' math pathways. *The Journal of Early Adolescence*, 29(1), 142-169. |
| 5 | Lindfors, P., Minkkinen, J., Rimpelä, A., & Hotulainen, R. (2018). Family and school social capital, school burnout and academic achievement: a multilevel longitudinal analysis among Finnish pupils. *International Journal of adolescence and Youth*, 23(3), 368-381. |
| 6 | Marjoribanks, K. (1994). Sibling and environmental correlates of adolescents' aspirations: family group differences. *Journal of Biosocial Science*, 26(3), 301-309. |
| 7 | Martin, M. A. (2012). Family structure and the intergenerational transmission of educational advantage. *Social Science Research*, 41(1), 33-47. |
| 8 | Devor, C. S., Stewart, S. D., & Dorius, C. (2018). Parental Divorce, Social Capital, and Postbaccalaurate Educational Attainment Among Young Adults. *Journal of Family Issues*, 39(10), 2806-2835. |
| 9 | Jhang, F. H., & Lee, Y. T. (2018). The role of parental involvement in academic achievement trajectories of elementary school children with Southeast Asian and Taiwanese mothers. *International Journal of Educational Research*, 89, 68-79. |
| 10 | Chiang, Y. L., & Park, H. (2015). Do grandparents matter? A multigenerational perspective on educational attainment in Taiwan. *Social Science Research*, 51, 163-173. |
